# Age-matched versus non-age-matched comparison of clinical and functional differences between delusional disorder and schizophrenia: a systematic review

**DOI:** 10.3389/fpsyt.2023.1272833

**Published:** 2023-10-10

**Authors:** Christy Lai Ming Hui, Tsz Ching Chiu, Evie Wai Ting Chan, Priscilla Wing Man Hui, Tiffany Junchen Tao, Yi Nam Suen, Sherry Kit Wa Chan, Wing Chung Chang, Edwin Ho Ming Lee, Eric Yu Hai Chen

**Affiliations:** ^1^Department of Psychiatry, School of Clinical Medicine, Li Ka Shing Faculty of Medicine, The University of Hong Kong, Pokfulam, Hong Kong SAR, China; ^2^State Key Laboratory of Brain and Cognitive Sciences, The University of Hong Kong, Pokfulam, Hong Kong SAR, China

**Keywords:** psychopathology, psychotic disorders, symptoms, cognition, functioning, outcomes, systematic review

## Abstract

**Background:**

It has been widely suggested that delusional disorder (DD) differs from schizophrenia (SZ). However, whether the two disorders are truly distinct from each other is inconclusive as an older age of onset is closely linked to a better prognosis in psychotic disorders. In order to delineate the potential influence of age on outcomes, we undertook a systematic review on the clinical and functional differences between DD and SZ in age-matched and non-age-matched cohorts.

**Methods:**

Electronic databases were retrieved up to May 2022. Included studies were analyzed with reference to statements about clinical, cognitive and functional differences between DD and SZ.

**Results:**

Data synthesized from 8 studies showed (1) extensive effects of age on positive, general psychopathological symptoms and functioning, but (2) consistent differences between the two disorders in terms of negative symptoms and hospitalizations regardless of age matching.

**Conclusion:**

There is currently insufficient evidence to conclude whether DD is completely distinct from SZ, but our review showed support for the confounding effect of age in comparisons of psychotic disorders with different ages of onset. Future studies shall take note of other possible confounding variables, methods of age-matching and the importance of longitudinal information in deducing whether the two disorders differ from each other in course and outcome.

## 1. Background

Kraepelin (1) first described “paranoia” as a chronic illness characterized by well-organized delusions in the absence of hallucinations while applying “paraphrenia” to schizophrenia (SZ) patients who experienced hallucinations in addition to delusions. Subsequently, Winokur (2) defined delusional disorder (DD) as non-bizarre delusions without accompanying hallucinations. Currently, DSM-V defines DD as per the presence of one or more delusions lasting for at least 1 month in the absence of prominent affective symptoms. Any hallucinations present must not be prominent, nor should patients appear odd or report functional impairments beyond the behavioral ramifications of their delusions.

The nosology of DD from other psychotic disorders such as SZ has always been of major interest in DD literature. A classic review of 17 studies (3) suggested that compared to paranoid psychosis, DD was characterized by an older age of onset, a shorter hospitalization, a greater number of females, married, non-foreign-born patients and slightly greater social disadvantages. Later studies reported similar findings in addition to better social functioning in DD (4). Until only recently, however, few studies have examined whether DD and SZ are separate entities (5, 6). This is potentially due to DD only making up around 0.03–0.18% of the general population and 0.4–4% of the hospital population (7). Features of the disorder such as high functioning and lack of insight may further limit the recruitment of an optimal sample size (8).

In the three decades since Kendler’s review (3), there has only been one longitudinal study comparing 43 patients with DD to 42 patients with paranoid SZ – although only 26 pure DD and 38 SZ patients remained after 12.9 years (5). In addition to confirming their many dissimilarities in symptoms, course and outcomes, including better social and functional outcomes in DD patients, DD was also found to be influenced more by environmental factors than genetics compared to SZ. Therefore, evidence has generally been in favor of differentiating between SZ and DD amongst the few existing studies in the area.

However, whether DD is truly distinct from SZ remains even more inconclusive because of existing biases in study samples. Notably, studies by Marneros et al. (5) and Jager et al. (4) included only inpatients in their cohort, which may pose issues such as sample representation. More importantly, neither study matched for age despite that DD is associated with an older age of onset. Psychotic symptoms during adolescence may have a more far-reaching detrimental effect on social and work functioning than if presented in later life considering that older patients are more likely to have better established careers and social networks (9). Indeed, an older age of onset has been closely linked to not only a better prognosis, but also compensates for symptoms prior to treatment (10, 11).

We previously attempted to provide empirical data on the issue of whether SZ encompasses a broad spectrum of or represents a separate disorder from other non-affective psychoses in an age-matched cohort (6). The cross-sectional comparison between 71 pairs of outpatients with adult-onset DD and SZ found that DD patients were more likely to be married and had less premorbid schizoid and schizotypal traits than SZ. Interestingly, no significant differences were found between the age-matched DD and SZ groups in terms of symptoms severity, functioning and neurocognitive performance. Therefore, it is crucial to pinpoint the potential confounding effect of age in order to address whether DD is truly distinct from SZ.

No reviews to date have examined the differences between comparing age and non-age-matched DD and SZ cohorts. To address this literature gap, this paper aimed to systematically review clinical, cognitive and functional differences between DD and SZ in age-matched as well as non-age-matched samples. We hypothesize that there may be differential outcomes when the moderating effects of age is taken into account.

## 2. Methods

### 2.1. Search strategy

Electronic searches were performed using the online databases of PsycINFO, Embase and PubMed from inception to 5th May 2022. Search terms are detailed in Table 1. Reference lists of relevant publications were manually checked to identify potential studies related to DD and SZ.

**Table 1:** Search terms applied in literature search.

PubMed and Embase shared the same set of search terms: (delusional disorder) AND ((schizophrenia) OR (psychosis) OR (psychotic disorders)) AND ((clinical) OR (cognitive) OR (cognition) OR (functioning)) while the following search strategy was used for PsycINFO: NOFT(delusional disorder) AND (NOFT(schizophrenia) OR NOFT(psychosis) OR NOFT(psychotic disorders)) AND (NOFT(clinical) OR NOFT(cognitive) OR NOFT(cognition) OR NOFT(functioning)

**Figure 1:**
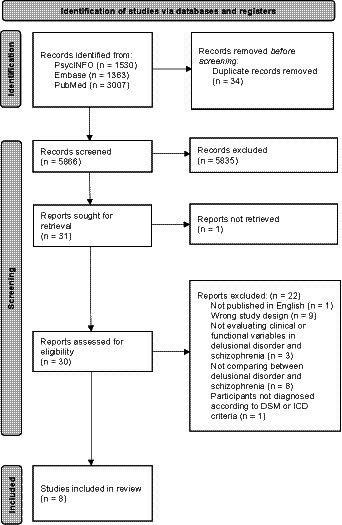
PRISMA flow diagram of included studies.

This yielded 5,900 records. Subsequent to the removal of duplicates, studies were screened for eligibility by titles and abstracts, and then by full texts (Figure 1). The search strategy was performed by three independent authors (LC, PH and CH). Any disagreement among the authors was resolved through discussions.

### 2.2. Inclusion and exclusion criteria

Articles were included if they met the following criteria: (1) included comparative data in the clinical, cognitive or functioning outcome of DD and SZ; (2) consisted of patients with DD and SZ according to ICD or DSM criteria; and (3) published in an English, peer-reviewed journal.

Articles were excluded if they were: (1) case reports, systematic reviews, protocols, conference abstracts, commentary or meta-analyses; (2) not comparing between DD and SZ; or (3) included patients without a clear description of the diagnostic criteria for DD and SZ according to the ICD or DSM.

### 2.3. Data collection and analysis

Titles and abstracts of retrieved publications were reviewed by three independent authors (LC, PH and CH) to determine relevance. Where titles and abstracts failed to provide sufficient indication of relevance, full articles were examined for eligibility with regards to the abovementioned inclusion and exclusion criteria. Studies included in the systematic review were then analyzed with reference to statements about clinical, cognitive or functional differences between DD and SZ.

### 2.4. Recorded variables and data synthesis

For each included study, the following variables were recorded: authors and year of publication, title, objectives, study design, study setting, location of study, participants’ age, onset age, diagnosis and its change over time and the outcome measures used. Main findings on the clinical, cognitive or functional outcomes between DD and SZ were presented separately for non-age-matched and age-matched samples, if available.

### 2.5. Risk of bias and quality assessment

For cross-sectional studies, the Joanna Briggs Institute (JBI) Critical Appraisal Checklist for analytical cross-sectional studies was used (12). One out of eight items were removed because of its irrelevance to the studies included (Table 2). The appraisal tool assessed the methodological quality of a study and addressed the possibility of bias in its design, conduct and analysis based on sample inclusion criteria, study setting, condition measurement, confounding factors, outcome measurement and statistical analysis. Each item was addressed with “Yes,” “No” or “Unclear.”

**Table 2:** Quality assessment for cross-sectional studies (JBI Critical Appraisal Checklist for analytical cross-sectional studies) and longitudinal studies (JBI Critical Appraisal Checklist for cohort studies).

Authors and year of publication	Sample inclusion criteria clearly defined?	Study subjects and the setting described?	Objective, standard criteria used for measurement of the condition?	Confounding factors identified?	Strategies to deal with confounding factors stated?	Outcomes measured in a valid and reliable way?	Appropriate statistical analysis used?	Follow up time reported and long enough for outcomes to occur?	Follow up complete or were reasons to loss to follow up described?	Strategies to address incomplete follow up utilized?	% yes	Risk
**Cross-sectional studies**												
Evans et al., 1996	Yes	Yes	Yes	Yes	Yes	Yes	Yes	/	/	/	100%	Low
Hui et al., 2015	Yes	Yes	Yes	Yes	Yes	Yes	Yes	/	/	/	100%	Low
Muñoz-Negro et al., 2015	Yes	Yes	Yes	No	No	Yes	Yes	/	/	/	71%	Low
Muñoz-Negro et al., 2017	Yes	Yes	Yes	No	No	Yes	Yes	/	/	/	71%	Low
Muñoz-Negro et al., 2018	Yes	Yes	Yes	Yes	Yes	Yes	Yes	/	/	/	100%	Low
Peralta and Cuesta, 2016	Yes	Yes	Yes	Yes	Yes	Yes	Yes	/	/	/	100%	Low
**Longitudinal studies**												
Marneros et al., 2012	/	/	/	Yes	No	Yes	Yes	Yes	No	Yes	71%	Low
Yassa and Suranyi-Cadotte, 1993	/	/	/	No	No	Unclear	Yes	Yes	No	No	29%	High

For cross-sectional studies, the Joanna Briggs Institute (JBI) Critical Appraisal Checklist for analytical cross-sectional studies was used (13). One out of 8 items was removed because the question was not relevant to the studies included. The appraisal tool assessed the methodological quality of a study and addressed the possibility of bias in its design, conduct and analysis based on sample inclusion criteria, study setting, condition measurement, confounding factors, outcome measurement and statistical analysis. Each item was addressed with “Yes,” “No” or “Unclear.” For longitudinal studies, the Joanna Briggs Institute (JBI) Critical Appraisal Checklist for cohort studies was adopted (14). Four out of 11 items were removed since the questions were not relevant to the studies included. The checklist assessed the methodological quality of a study and addressed the possibility of bias in its design, conduct and analysis based on confounding factors, outcome measurement, statistical analysis and follow up strategies. Each item was addressed with “Yes,” “No” or “Unclear.” For both types of studies, the risk of bias was ranked as high when “yes” scores were ≤ 49%, moderate when “yes” scores were between 50 and 69%, and low when “yes” scores were above 70%.

For longitudinal studies, the JBI Critical Appraisal Checklist for cohort studies was adopted (15). Four out of 11 items were removed since the questions were not relevant to the studies included (Table 2). The checklist assessed the methodological quality of a study and addressed the possibility of bias in its design, conduct and analysis based on confounding factors, outcome measurement, statistical analysis and follow up strategies. Each item was addressed with “Yes,” “No” or “Unclear.” For both types of studies, the risk of bias was ranked as high when “yes” scores were ≤ 49%, moderate when “yes” scores were between 50 and 69% and low when “yes” scores were above 70%.

## 3. Results

### 3.1. Study selection

Of the 5,900 articles initially retrieved, 1,530 were identified in PsycINFO, 1,363 in Embase and 3,007 in PubMed. Following title and abstract screening, 31 articles remained for full-text retrieval, one of which was excluded for lacking documentation of a full article. Of the remaining 30 articles, 22 were excluded: one was not published in English, nine were excluded due to study design, three did not evaluate clinical or functional variables in DD and SZ, eight did not compare between DD and SZ, and one included participants without a clear description of the diagnostic criteria for DD and SZ according to the ICD or DSM. In total, eight studies fulfilled our inclusion/exclusion criteria and were therefore included in the systematic review (Figure 1). Two of those studies shared the same sample pool (16, 17). Characteristics of the included studies were summarized and discussed in Table 3.

### 3.2. Study design and setting

Of the eight included studies, six were cross-sectional studies and two were longitudinal with a follow-up period of 13 years (5) and 7 years (18). Four studies were compiled and published in Spain, one in Canada, one in the United States, one in Germany and one in Hong Kong. Four out of eight studies recruited patients from outpatient clinics, three recruited from inpatient facilities and one recruited from a university medical center as well as the community.

### 3.3. Patients and diagnoses

Three of the eight studies compared between DD and SZ (6, 17, 19). Two studies compared between DD, SZ and schizoaffective disorder (16, 17). One study compared between DD, paranoid SZ and non-paranoid SZ (20), one study compared patients with late-onset SZ, DD with hallucination and DD without hallucination (18), and one study compared between DD and paranoid SZ (5). DD and paranoid SZ patients in the last study were diagnosed according to ICD and DSM criteria, while the diagnosis in all other studies was made according to DSM criteria. This comprised of a total of 585 DD patients, 1,124 SZ patients and 63 schizoaffective disorder patients.

### 3.4. Age-matched cohorts

Four studies conducted age-matched comparisons. Evans et al. (14) selected only patients with illness onset after aged 40 to produce an age-matched cohort. The mean onset age was 60.4 years for DD patients and 54 years for SZ patients.

Hui et al. (6) identified an age-matched cohort by propensity score matching, including DD patients with a mean age of 39.4 and SZ patients with a mean age of 39.1 at first episodes.

Two other studies (19, 20) performed age-matched comparisons between DD and SZ patients by statistical age-adjustment. In one study, the mean onset age was 38.8 for DD patients, 30.5 for paranoid SZ patients and 23.9 for non-paranoid SZ patients (20). The age of onset was not documented by Muñoz-Negro et al. (19).

Three of the four aforementioned studies conducted non-age-matched comparisons as well.

The remaining four studies (5, 16–18) performed only non-age-matched comparisons between non-matched DD and SZ cohorts.

### 3.5. Group differences in age-matched cohorts

#### 3.5.1. Group differences in clinical aspects

Out of the three studies that examined differences in positive symptoms between DD and SZ, Hui et al. (6) reported fewer hallucinations but insignificantly more delusions in DD. Peralta and Cuesta (20) reported less but more severe delusions in DD when compared to paranoid SZ and non-paranoid SZ, while Evans et al. (14) observed an insignificant trend of DD displaying more positive symptoms than SZ.

Two studies compared negative symptoms between DD and SZ, where both found an insignificant trend for DD to demonstrate less severe negative symptoms (6, 14).

Of the four studies that performed age-matched comparisons, three examined differences in general psychopathology between DD and SZ. Whilst two out of three studies reported DD having higher psychopathology ratings (6, 14), this trend was not significant in Hui et al.’s study (6). On the contrary, the third study reported DD with less severe psychopathology compared to SZ (19).

All three studies (6, 14, 20) that examined hospitalization in DD and SZ reported fewer hospitalizations in DD.

#### 3.5.2. Group differences in functioning and cognitive functioning

Results on social and occupational functioning between DD and SZ varied across studies. While one study did not see any difference on functioning between the two groups (6), Muñoz-Negro et al. (19) found that DD patients have better global functioning. Further, at one-year follow-up, Peralta and Cuesta (20) found that DD patients had *better* personal care, social functioning and having a higher number of paid work, but *poorer* occupational functioning.

Two studies that explored cognitive functioning between SZ and DD yielded insignificant findings. Although Evans et al. (14) found that neuropsychological impairment was generally lower in DD, this was not significant. Neither was the trend observed by Hui et al. (6), as the two groups performed similarly across a broad range of neurocognitive assessments.

#### 3.5.3. Group differences in demographics

Two studies compared gender differences, years of education and premorbid functioning between DD and SZ. Neither study found significant differences between the two groups (6, 14).

Results on marital status between DD and SZ, however, varied. While Evans et al. (14) reported DD patients as being less likely to be married, Hui et al. (6) found opposite results.

### 3.6. Group differences in non-age-matched cohorts

#### 3.6.1. Group differences in clinical aspects

Out of the six studies that examined differences in positive symptoms between DD and SZ, one did not find any group difference (17) while five reported positive symptoms to be less frequent in DD (5, 16, 18–20). Specifically, two studies found that first-rank symptoms did not occur in DD (5, 18). As for hallucinations, two studies reported DD as having fewer hallucinations when compared to SZ (5, 19).

With regard to delusions, two studies revealed no significant differences (5, 19) and one study reported DD as having less but more severe delusions (20). One study found SZ to be characterized by bizarre delusions and DD by non-bizarre delusions (18).

Findings on negative symptoms between DD and SZ were consistent across five studies. All studies reported less frequent negative symptoms in DD when compared to SZ (5, 16–19).

Of the seven studies that performed non-age-matched comparisons, three studies analyzed general psychopathology between DD and SZ. Two out of 3 studies (5, 19) reported DD with less severe psychopathology although Marneros et al. (5) indicated the trend to be insignificant. Meanwhile, Muñoz-Negro et al. (17) found no significant differences between DD and SZ.

Three studies examined hospitalization in DD and SZ. Marneros et al. (5) revealed DD as having less frequent hospitalizations and a shorter duration of their hospitalizations compared to paranoid SZ. Yassa and Suranyi-Cadotte (18) reported similar findings, but the results not reach statistical significance. When compared to both paranoid SZ and non-paranoid SZ, Peralta and Cuesta (20) also found DD to have fewer hospitalizations.

#### 3.6.2. Group differences in functioning and cognitive functioning

Results of the five studies comparing social and occupational functioning between DD and SZ were in agreement with each other, with all reporting DD to have better functioning. Specifically, two studies reported better global functioning (16, 19) and one study reported more full-time employment in DD (6). Another study reported DD patients as being more likely to be employed and less likely to retire early as well as having lower scores in the Disability Assessment Scale when compared to paranoid SZ patients (5). The final study reported DD to be associated with better personal care and social functioning and a higher number of paid work, but poorer occupational functioning than paranoid and non-paranoid SZ at one-year follow up (20).

Of the seven studies that performed non-age-matched comparisons, only one study explored cognitive functioning in SZ and DD (6). In line with the age-matched comparison within the study, Hui et al. (6) revealed that neurocognitive performance was not significantly different between non-matched DD and SZ cohorts.

#### 3.6.3. Group differences in demographics

Of the seven studies that conducted non-age-matched comparisons, three studies compared onset age differences between DD and SZ with all reporting DD as having an older age of onset (5, 18, 20).

Six studies compared gender differences between DD and SZ. Despite that three studies found no gender differences between DD and SZ (6, 17, 20), three other studies reported a higher prevalence of women among DD patients (17–19).

Of the six studies that explored education differences, four studies reported insignificant differences between DD and SZ (5, 17–19), one study noted that incomplete primary studies were more frequent among DD patients whilst complete higher studies were more frequent among SZ patients (16) and one study reported DD as having less years of education than paranoid and non-paranoid SZ patients (20).

Furthermore, three studies examined marital status among DD and SZ patients. Two demonstrated a higher frequency of marriage in DD (19, 20) while one (18) found no difference in marital status between the two disorders.

### 3.7. Diagnostic change over time

Two out of eight studies documented diagnostic change over time. Over the follow-up period of up to 8 years, none of the DD or SZ patients had a change in their primary clinical diagnosis (14). Meanwhile, another study recorded 21.2% of the DD patients shifting into SZ or schizoaffective disorder during a period of 10.8 years (5). The remaining 78.8% of DD patients had no syndrome shift.

**Table 3:** Characteristics of included studies.

Study	Study design	Study setting	Participants - diagnoses	Participants - age	Participants - onset age	Outcomes	Key results - age-matched	Key results - non-age-matched
Evans et al., 1996 (14)	Cross-sectional	California United States; From the University of California Medical Center and the Community	Out of 14 DD + 253 SZ (DSM-III), aged-matched cohort of 14 DD + 50 SZ were used for comparison (the cohort is aged-matched by selecting only patients with illness onset after age 40)	Age-matched samples: DD: 66.9 (13.6) years SZ: 63.5 (8.9) years	Age-matched samples: DD: 60.4 (13.9) years SZ: 54 (9.7) years	• Clinical: BPRS, SAPS, SANS, HAMD, G-K (on premorbidity), AIMS •Neuropsychological: Attention, Verbal, Motor, Psychomotor, Learning, Memory, Abstraction, Sensory	Age-matched patients (onset after the age of 40): • DD: greater psychopathology (on BPRS); insignificant trend of fewer negative symptoms, fewer hospitalizations, lower daily neuroleptic doses • DD: lower neuropsychological impairment but not significantly • DD: less likely to be married; no significant difference in gender, years of education, premorbid adjustment (on G-K)	/
Hui et al., 2015 (6)	Cross-sectional	Hong Kong; From outpatient psychiatric units at an early intervention clinic (the Jockey Club Early Psychosis (JCEP) Project)	Out of 72 first episode DD + 157 first episode SZ (DSM-IV), aged-matched cohort of 71 DD + 71 SZ were used for comparison (propensity score matching)	Age-matched samples: DD: 41.8 (8.3) years SZ: 40.8 (8.7) years	Age-matched samples: DD: 39.4 (8.7) years SZ: 39.1 (9.3) years	• Premorbid and help-seeking characteristics: PAS, PSST • Clinical: hospitalization, comorbidities, medical illness, PANSS, SAPS, SANS, antipsychotic medication • Functioning: SOFAS, RFS • Cognitive: information, arithmetic, digit symbol, VPT, digit span, logical memory, verbal fluency	Age-matched patients: • DD: fewer hallucination (on SAPS), insignificantly more delusions (on SAPS), fewer hospitalizations, more psychiatric comorbidities (affective disorder); no difference in psychopathology (on PANSS) • No significant differences in social and occupational functioning and neurocognitive performance • DD: less premorbid schizoid and schizotypal traits (thought content), more likely to be married; no significant difference in gender, education, premorbid adjustment (on PAS)	• Cognitive functioning and gender were not significantly different • DD: more full-time employment
Marneros et al., 2012 (5)	Prospective, longitudinal follow-up of an average of 13 years following onset	Germany; From inpatient at the Department of Psychiatry, Psychotherapy and Psychosomatics at the Martin Luther University	43 DD (DSM-IV and ICD-10) + 42 PSZ (DSM-IV)	Age at index admission: DD: 51.8 (12.6) years PSZ: 41.1 (12.4) years	DD: 46.9 (13.2) years PSZ: 35.3 (13.9) years	• Clinical: PANSS • Functioning: SOFAS, GAF, DAS	/	• DD: less severe psychopathology but not significant; no first-rank symptoms, primary hallucinations, or relevant negative symptoms; no difference in delusion; less frequent and shorter hospitalization • DD: better employment, fewer early retirement due to the disorder, fewer on psychopharmacological medication; more often autarkic (living independently); lower scores in the DAS • DD: an older age of onset, broken home background; no significant difference in education.
Muñoz-Negro et al., 2015 (16)	Observational; the study combined data from 5 independent studies using compatible and similar assessment methods	Spain; From psychiatric outpatient clinics	550 psychotic disorders (373 SZ + 137 DD + 40 SA) (DSM-IV)	DD: 49.8 (14.7) years SZ: 35.9 (13.1) years SA: 46.7 (14.4) years	/	• 5 dimensions (manic, negative, depression, positive, cognitive) derived from PANSS and GAF measures	/	• DD had less positive and negative psychotic symptoms lower negative, cognitive dimensions; lower positive dimension (intermediate in SZ, higher in SA); depressive and manic dimensions higher among SA • DD had higher global functioning (lower in SZ, intermediate in SA); no gender differences but more males within SZ; more frequent incomplete primary studies, whilst complete higher studies were more frequent among SZ patients
Muñoz-Negro et al., 2017 (17)	Observational	Spain; From outpatient department at different hospitals and community mental health settings	112 psychotic disorders (67 SZ + 22 DD + 23 SA) (DSM-IV)	DD: 49.6 (12.6) years SZ: 40.4 (11.5) years SA: 44.4 (13.4) years	/	PANSS, Premorbid IQ, educational level	/	• No difference in general psychopathology, positive symptoms; SA had more severe positive symptoms than DD and SZ; SA and SZ had more severe negative symptoms than DD • No gender difference between DD and SZ; premorbid IQ and years of education were not significantly different between DD, SZ and SA
Muñoz-Negro et al., 2018 (19)	Cross-sectional comparisons, the study combined data from 3 independent studies, including both Muñoz-Negro et al. (16, 17)	Andalusia and Catalonia, Spain; From psychiatric outpatient clinics (public or private mental health services integrated or commissioned by the Spanish National Health Service)	275 patients (132 DD + 143 SZ) (DSM-IV)	DD: 50.3 (14.6) years SZ: 36.6 (11.1) years	/	•Sociodemographics (marital status, premorbid IQ, employment status, educational level) • Clinical: PANSS • Functioning: GAF	Age-adjusted patients: • DD: less severe psychopathology (on PANSS), better global functioning	On crude analysis: • DD: less severe psychopathology (on PANSS), fewer positive, negative symptoms, hallucination; no significant difference in delusion • DD: better global functioning, less work-related disability • DD: older, more frequently married; had higher estimated premorbid IQ; no gender difference in DD but more males in SZ
Peralta and Cuesta, 2016 (20)	Cross-sectional study with 1 year fup	Spain; From inpatient at the Virgen del Camino Hospital	146 DD + 114 PSZ + 244 NPSZ (DSM-IV)	DD: 49.4 (15.0) years PSZ: 40.0 (15.7) years NPSZ: 34.5 (13.1) years (DD > PSZ > NPSZ)	DD: 38.8 (14.3) years PSZ: 30.5 (13.4) years NPSZ: 23.9 (8.54) years (DD > PSZ > NPSZ)	• CASH (premorbid, SAPS, mood disorders) • 1-year fup functioning: personal care, occupation, household, social context, paid work, GAF	Age-adjusted patients: • DD: less but more severe delusions especially on jealousy, higher conviction and lower disorganization of delusional experiences, higher likelihood of major depression, chronic illness course, lack of insight, less hospitalizations • At 1-year fup, DD: better personal care and social functioning, higher numbers of paid work, poorer occupational functioning • DD: older onset age	• Of 52 variables, 40 differentiated DD from PSZ and/or NPSZ; 29 differentiated DD from both SZ, 9 differentiated DD from NPSZ, 2 differentiated DD from PSZ • PSZ was similar to NPSZ on 17 variables but similar to DD on only 7 • DD associated with the following clinical features: less but more severe delusions, especially on jealousy/somatic, higher conviction and lower disorganization of delusional experience, less hospitalization; more likelihood of major depression, higher index episode ratings of depressed mood, dysphoria, anxiety, chronic illness course, lack of insight, poorer responses to antipsychotic drugs • DD associated with the following psychosocial functioning features (at 1-year fup): better personal care and social functioning, higher numbers of paid work, poorer occupational functioning • DD associated with the following demographics: less years of education, more likely married, older, older onset age; no significant gender difference
Yassa and Suranyi-Cadotte, 1993 (18)	Longitudinal, 7-year observation period	Canada; From inpatient at the acute psychogeriatric unit	20 LOS + 7 DD with hallucinations +13 DD without hallucinations (DSM-III)	DD: 77.3 (7.2) years DD + H: 74.1 (3.8) years SZ: 78.7 (8.0) years	Age of first admission: DD: 71.3 (9.0) years DD + H: 58.9 (9.3) years SZ: 62.1 (10.7) years	• Clinical variables • Concomitant physical disorders	/	Clinical features: • LOS characterized by: bizarre delusions, AH, first-rank and negative symptoms, premorbid paranoid/schizoid personality • DD associated with: non-occurrence of negative symptoms, non-bizarre delusions, late onset of symptoms, relatively intact premorbid personality, underlying physical stratum, fewer hospitalizations and shorter duration of hospitalization but difference was insignificant • DD + H associated with: non-bizarre delusions, AH, earlier onset of symptoms, premorbid paranoid/schizoid personality Demographics: • DD: older age of onset, higher prevalence of women; no significant differences in education level and marital status

“/”, not applicable; Fup, follow-up; DD, delusional disorder; SZ, schizophrenia; SA, schizoaffective disorder; PSZ, paranoid schizophrenia; NPSZ, non-paranoid schizophrenia; LOS, late-onset schizophrenia; H, hallucinations; AH, auditory hallucination; BPRS, Brief Psychiatric Rating Scale; SAPS, Scale for the Assessment of Positive Symptoms; SANS, Scale for the Assessment of Negative Symptoms; HAMD, Hamilton Depression Rating Scale; G-K, Gittelman-Klein Premorbid Social Adjustment Scale; AIMS, Abnormal Involuntary Movement Scale; PAS, Premorbid Adjustment Scale; PSST, Assessment of Premorbid Schizoid and Schizotypal Traits; PANSS, Positive and Negative Syndrome Scale; SOFAS, Social Occupational Functioning Scale; RFS, Role Functioning Scale; VPT, Visual Patterns Test; GAF, Global Assessment of Functioning; DAS, Disability Assessment Scale; CASH, Comprehensive Assessment of Symptoms and History.

## 4. Discussion

This is the first systematic review to compare DD and SZ in age-matched and non-age-matched cohorts. Eight studies were included to evaluate the clinical, cognitive and functional differences between DD and SZ. DD was found to have *less* severe positive and general psychopathology symptoms in studies that did not control for age. But consistently across age-matched and non-age-matched cohorts, DD had fewer negative symptoms, better functioning and fewer hospitalizations. Though no differences in cognitive functioning, gender, education and premorbid functioning were observed, DD was more likely to be married in both age-matched and non-age-matched comparisons. While it remains questionable whether DD and SZ are separate entities, our systematic review reveals consistent findings across age-matched and non-age-matched analyses on a number of variables. It is also pertinent to note the effect of age on outcomes such as clinical variables and occupational functioning.

### 4.1. Age effect on positive and general psychopathology symptoms

The effect of age was apparent on positive and general psychopathology symptoms, but absent for negative symptoms, functioning and hospitalizations. Regarding positive symptoms and general psychopathology, DD patients were found to have more severe symptoms in age-matched cohorts but less severe symptoms in non-age-matched cohorts when compared to SZ patients. With existing research suggesting younger age to be associated with a poorer prognosis in SZ (10, 11), one may reasonably expect DD patients who are generally older (3, 4) to have less severe positive and general psychopathology symptoms than SZ patients in non-age-matched comparisons.

However, the effect of age on negative symptoms and hospitalization remains ambiguous. In accordance with other studies indicating DD patients to be characterized by less pronounced negative symptoms like flat affect and alogia (4), we found the DD displayed less severe negative symptoms irrespective of age-matching. This may be because studies that included only outpatients were biased towards clinically less severe samples, particularly in DD patients, leading to more notable differences between DD and SZ. Though unable to conclude DD and SZ as completely separate entities, our results reiterated dissimilarities between the two groups and suggested the possibility of a psychopathological gradient regarding negative symptoms among psychotic disorders.

Similarly, we found DD patients to have fewer hospitalizations regardless of age-matching. Existing studies that reported non-age-matched DD cohorts as having fewer hospitalizations may reflect the observation of a better prognosis for DD patients who tend to be older (5, 13). Our consistent findings across age-matched and non-age-matched studies, however, challenged this explanation considering the minimal effect of age on hospitalizations. The fact that most DD patients were hospitalized due to social reasons (5) may imply that they were less disturbed by clinical symptoms in the first place, accounting for fewer hospitalizations in general. It should also be noted that most of the existing studies did not explore reasons of hospital admissions. It would therefore be worthy to compare reasons of admissions such as relapse, suicide, or comorbid health conditions. Moreover, very few studies looked at voluntary and involuntary admissions. Further investigation on the types of hospital admission and its relationship with help-seeking behaviors or insight would be meaningful. Despite the absence of age effect on negative symptoms and hospitalizations, the inconsistent findings regarding positive and general psychopathology symptoms reveal how the effect of age on clinical characteristics remains pivotal.

### 4.2. Age effect on functioning

Given that an older age of onset was closely linked with better prognosis in psychotic disorders (10, 11), it is reasonable to expect aged- and non-age-matched differences in functioning outcomes between DD and SZ.

In non-age-matched samples, our results consistently pointed towards better global, social and occupational functioning in DD patients. This observation was in line with our expectation that in comparison to their SZ counterparts, DD patients would be more likely to manifest better functioning due to their older age.

However, further investigation into studies that compared both aged-matched and non-aged-matched cohorts may provide more important clues as to the impact of age on these outcomes. For instance, Hui et al. (6) found better occupational outcome in DD in non-aged-matched analyses, but the same study did not observe such a difference when patients were matched by age, implying the substantial impact age has on functioning outcomes. Meanwhile, Peralta and Cuesta (20) found age-matched and non-age-matched DD patients to consistently demonstrate better functioning, potentially due to the age adjustment method adopted.

As for cognitive functioning, both age-matched and non-age-matched studies consistently found no difference between DD and SZ. However, it should be noted that only two studies looked at neurocognitive functioning, rendering insufficient data in concluding with certainty that DD and SZ do not differ from each other in terms of cognitive functioning.

### 4.3. Age effect on gender, education and premorbid functioning

While age influences gender, education and premorbid functioning between the two groups, it has little to no effect on marital status. In line with previous studies that reported DD patients with less deterioration of social, intimate and established relationship before onset (3, 4, 21), we found that more DD patients were married regardless of age-matching. Additionally, the mean age of onset for DD patients in our included studies was above 40. Since the illness occurred during middle-to-late adult life, it is more likely for DD patients to have been married by the time they fell ill. The consistent findings across age-matched and non-age-matched studies thus diminished the effect of age on marital status.

Nevertheless, we observed inconsistent results for other demographic variables. While DD patients were found to be less educated and were predominated by women in non-age-matched cohorts, no differences in gender, education and premorbid functioning were recorded in age-matched cohorts. In view of the discrete results, it is plausible to speculate an effect of age regarding the aforementioned variables.

### 4.4. Limitations

While the consistency of findings was generally good across studies, it was difficult to conclude the relative impairment between DD and SZ in several of the outcomes. For instance, ratings of general psychopathology between age-matched cohorts tended to be higher for DD in two studies (6, 14), but the opposite was observed in another (19). This may be related to the large discrepancies between the studies reviewed, as not all had matched for age. Further, some of the non-age-matched studies recruited only inpatients (5, 18, 20), some combined samples from five independent studies (16, 19) and one recruited DD and SZ patients at slightly different periods (20). That the majority of the samples were not truly representative makes age-matching of paramount importance. Additionally, longitudinal studies are crucial in identifying the distinctions between DD and SZ in the long term, but the two included were both non-age-matched and recruited only inpatients. They only provided enough information to conclude DD and SZ inpatients to be distinct from each other when not matched for age.

Furthermore, methods of age-matching varied across studies. One study conducted propensity score matching (6) while another selected only patients with illness onset after age 40 (14), which may have led to fewer SZ cases in the sample. Some performed statistical age adjustments (19, 20) which may not have yielded accurate measures of actual differences. Given the above discrepancies, a truly representative sample accurately matched by age may be needed for comparison between DD and SZ.

The directness of evidence is also limited by the discrepancies in patient groups across studies. For instance, some studies only involved patients with DD and PSZ (4) while others also included SA groups in their comparisons (16, 17). As previously mentioned, some of the studies also only recruited inpatients while others only outpatients. Recruiting DD inpatients may create a bias towards admission due to functional reasons instead of sheer clinical symptoms (5), with a greater severity of symptoms across all inpatients. Therefore, outpatients should also be included to secure a more representative sample of DD, especially given the questionable accuracy of hospital admission data regarding the true occurrence of DD in the population (3).

Further detracting from directness is the discrepancies in outcomes measures between studies. While most of the studies assessed clinical characteristics using the Positive and Negative Syndrome Scale, one study (20) adopted the Comprehensive Assessment of Symptoms and History. Another study (18) did not indicate the clinical scales used in their assessment. Assessment materials for functioning also varied across studies, with some adopting Social and Occupational Functioning Assessment Scale, some using Global Assessment of Functioning and some Disability Assessment Scale.

Similarly, diagnostic classification systems varied across studies. While the majority of the studies made diagnosis according to DSM criteria, one study had patients diagnosed according to ICD and DSM criteria. Additionally, while most of the studies that adopted DSM criteria diagnosed according to DSM-IV, two opted for DSM-III which may now be considered outdated. Using uniform and updated diagnostic classification systems shall thus be useful to ensure diagnostic categorizations are met.

Multiple comparisons were not always controlled for in the included studies. Some studies (6, 17) took the problem of multiple testing into account, but some (17, 20) did not. This might inflate the possibility of Type I errors which may cause an overestimation of existing differences between DD and SZ.

With the primary focus being on English literature, not all available data pertaining to the topic may have been identified. Conclusions about the differential outcomes of DD and SZ may consequently be underestimated especially when applied to non-Western countries. Further selection bias may have been introduced by including three studies that were conducted by the same authors (16, 17, 19), in which one (19) partially derived their data from the other two independent studies (16, 17). Conclusions about the quality of the studies reviewed may also be limited as the relevant authors were not contacted for clarifications, despite one study neglecting to state the materials they used to measure clinical outcomes (18).

### 4.5. Clinical and research implications

Neurobiological research into the cellular and molecular mechanisms underlying psychotic disorders may provide additional insight about the nosologies of DD and SZ. Previously, gray matter reductions in the superior temporal gyrus and cerebellum were indicated as neuroanatomical markers of psychosis (22). Future research may be guided by the Research Domain Criteria project to identify genes, cells, and other units of analysis associated with the superordinate functional constructs of psychotic disorders, such as negative and positive affect, cognition, and social processes (23). In this way, neurobiological advances may help to further refine diagnostic classification beyond observable characteristics, and better account for the outcomes of DD and SZ.

As of now, current evidence suggests that DD and SZ demonstrate similar levels of cognitive impairments regardless of age. Cognitive treatments that have recently been recommended for SZ (24) may thus also be applicable to improving the cognitive performance of patients with DD. Of particular relevance is cognitive remediation therapy, which offers benefits across different cognitive domains including memory, planning, problem solving and social cognition, independently of age (25).

### 4.6. Current and future directions

There is currently insufficient evidence to conclude whether DD is completely distinct from SZ. Our systematic review has found extensive effect of age on positive and general psychopathological symptoms as well as functioning, but consistent differences between DD and SZ in terms of negative symptoms and hospitalizations regardless of age matching. From these we can only infer that DD and SZ exhibit dissimilarities regarding negative symptoms and hospitalizations at the time of data collection. Moreover, since too few studies explored cognitive functioning, there is insufficient empirical data to determine whether SZ and DD patients differ from each other in terms of cognition. Another reason for the evasive conclusion would be due to difficulty in ascertaining enough DD sample. Given DD accounting for less than 1% of hospital admissions (3), studies that recruited inpatients only might end up with a small DD sample size. Also, many studies did not control for multiple comparisons, therefore existing differences between DD and SZ might be overestimated.

Our systematic review supports that age is an important prognostic factor in SZ, future studies should thus bear in mind the confounding effect of age when comparing different psychotic disorders with different ages of onset. As mentioned above, whether statistical age adjustments are accurate measures of actual differences remains questionable and that the selection of patients with older onset age would bias towards fewer SZ cases. When DD and SZ patients of all age groups are recruited during a particular period for comparison, the problem of neglecting adolescent-onset cases would be minimized. Alternatively, future studies should at least include outpatients to ascertain a more representative DD sample, while making sure to collect longitudinal information which is crucial to determine whether DD is distinct from SZ in terms of course and outcome in the long term. Future studies should take into account other confounding variables such as cultural differences. For instance, there is lower cannabis use in Asia (<0.5%) than in the Western countries (>10%) (26), thereby influencing risk factors such as substance abuse before onset. Also, the fact that Chinese population who suffers from serious mental illness tend to demonstrate more self-blame (27), might give rise to reduced openness domestically and less prevalent professional help seeking behaviors.

## 5. Conclusion

Despite insufficient evidence to conclude whether DD is completely distinct from SZ, our review showed support for the confounding effect of age in the comparisons of psychotic disorders with different ages of onset. This review also better informs the differential clinical categorization of DD and SZ by taking age into account. Overall, DD was generally associated with better psychopathological and functioning outcomes regardless of age. However, neglecting age from considerations may lead to misinterpretations as positive and general psychopathology were only less severe for DD patients when the current cohorts were age-matched.

## Data availability statement

The raw data supporting the conclusions of this article will be made available by the authors, without undue reservation.

## Author contributions

CH: Conceptualization, Formal analysis, Funding acquisition, Investigation, Methodology, Project administration, Writing – original draft, Writing – review & editing. TC: Conceptualization, Formal analysis, Investigation, Methodology, Writing – original draft, Writing – review & editing. EvC: Writing – review & editing. PH: Writing – review & editing. TT: Writing – review & editing. YS: Writing – review & editing. SC: Writing – review & editing. WC: Writing – review & editing. EL: Writing – review & editing. ErC: Writing – review & editing.

## Glossary

AIMS: Abnormal Involuntary Movement Scale
AH: Auditory hallucination
BPRS: Brief Psychiatric Rating Scale
CASH: Comprehensive Assessment of Symptoms and History
DAS: Disability Assessment Scale
DD: Delusional disorder
DSM: Diagnostic and Statistical Manual of Mental Disorders
Fup: Follow-up
GAF: Global Assessment of Functioning
G-K: Gittelman-Klein Premorbid Social Adjustment Scale
H: Hallucinations
HAMD: Hamilton Depression Rating Scale
ICD: International Classification of Diseases
JBI: Joanna Briggs Institute
JCEP: Jockey Club Early Psychosis
LOS: Late-onset schizophrenia
NPSZ: Non-paranoid schizophrenia
PANSS: Positive and Negative Syndrome Scale
PAS: Premorbid Adjustment Scale
PSST: Assessment of Premorbid Schizoid and Schizotypal Traits
PSZ: Paranoid schizophrenia
RFS: Role Functioning Scale
SA: Schizoaffective disorder
SANS: Scale for the Assessment of Negative Symptoms
SAPS: Scale for the Assessment of Positive Symptoms
SOFAS: Social Occupational Functioning Scale
SZ: Schizophrenia
VPT: Visual Patterns Test
